# Yttrium-90 glass microsphere radioembolization as frontline treatment for hepatocellular carcinoma with localized portal vein invasion

**DOI:** 10.1007/s00330-025-11882-w

**Published:** 2025-07-26

**Authors:** Jin Woo Choi, Minseok Suh, Yunhee Choi, Myungsu Lee, Jin Chul Paeng, Hyo-Cheol Kim

**Affiliations:** 1 Department of Radiology, Seoul National University Hospital, Seoul, Korea; 2 Department of Radiology, Seoul National University College of Medicine, Seoul, Korea; 3 Department of Nuclear Medicine, Seoul National University Hospital, Seoul, Korea; 4 Division of Medical Statistics, Medical Research Collaborating Center, Seoul National University Hospital, Seoul, Korea

**Keywords:** Radioembolization, Hepatocellular carcinoma, Portal vein invasion, Dosimetry

## Abstract

**Objectives:**

To evaluate the outcomes of yttrium-90 radioembolization (glass microspheres) in patients with unilobar hepatocellular carcinoma (HCC) and portal vein invasion (PVI) who have preserved liver function.

**Materials and methods:**

This study included 48 patients with unilobar HCC and PVI, all with Child-Pugh A, treated with radioembolization at a single institution between January 2016 and December 2023. Tumor response was assessed using the modified Response Evaluation Criteria in Solid Tumors (mRECIST) and localized mRECIST. Overall survival (OS) and prognostic factors were evaluated using time-to-event analyses. The mean tumor absorbed dose (TAD) threshold for achieving complete response (CR) by localized mRECIST was determined using receiver operating characteristic analysis, while the threshold associated with significantly longer OS was identified using the minimum *p*-value approach.

**Results:**

Objective response rates were 83% (40/48) by mRECIST and 88% (42/48) by localized mRECIST. The median OS was 47.2 months (95% CI, 19.1–52.1 months). The TAD was the only significant predictor of OS (*p* = 0.032, hazard ratio = 0.862 per 100 Gy, 95% CI = 0.753–0.988). A mean TAD > 574 Gy provided 50% sensitivity and 86% specificity for predicting CR by localized mRECIST, while a threshold of 586 Gy was proposed to significantly extend OS (median OS, 49.5 months for > 586 Gy and 21.9 months for ≤ 586 Gy; *p* = 0.021).

**Conclusion:**

Radioembolization is effective for HCC with localized PVI in patients with preserved liver function, and a mean TAD > 600 Gy is proposed to achieve improved oncologic outcomes.

**Key Points:**

***Question*** *What is the optimal radioembolization approach and its outcome for hepatocellular carcinoma with localized portal vein invasion (Vp1–3) in patients with preserved liver function?*

***Findings*** *A tumor absorbed dose exceeding 600 Gy via a tandem approach achieved complete response rates above 80% and median overall survival longer than 49.5 months.*

***Clinical relevance*** *Ablative radioembolization, delivering a tumor absorbed dose exceeding 600 Gy via a tandem approach, should be considered for hepatocellular carcinoma with localized portal vein tumor thrombosis (Vp1–3) in patients with preserved liver function and no extrahepatic spread.*

**Graphical Abstract:**

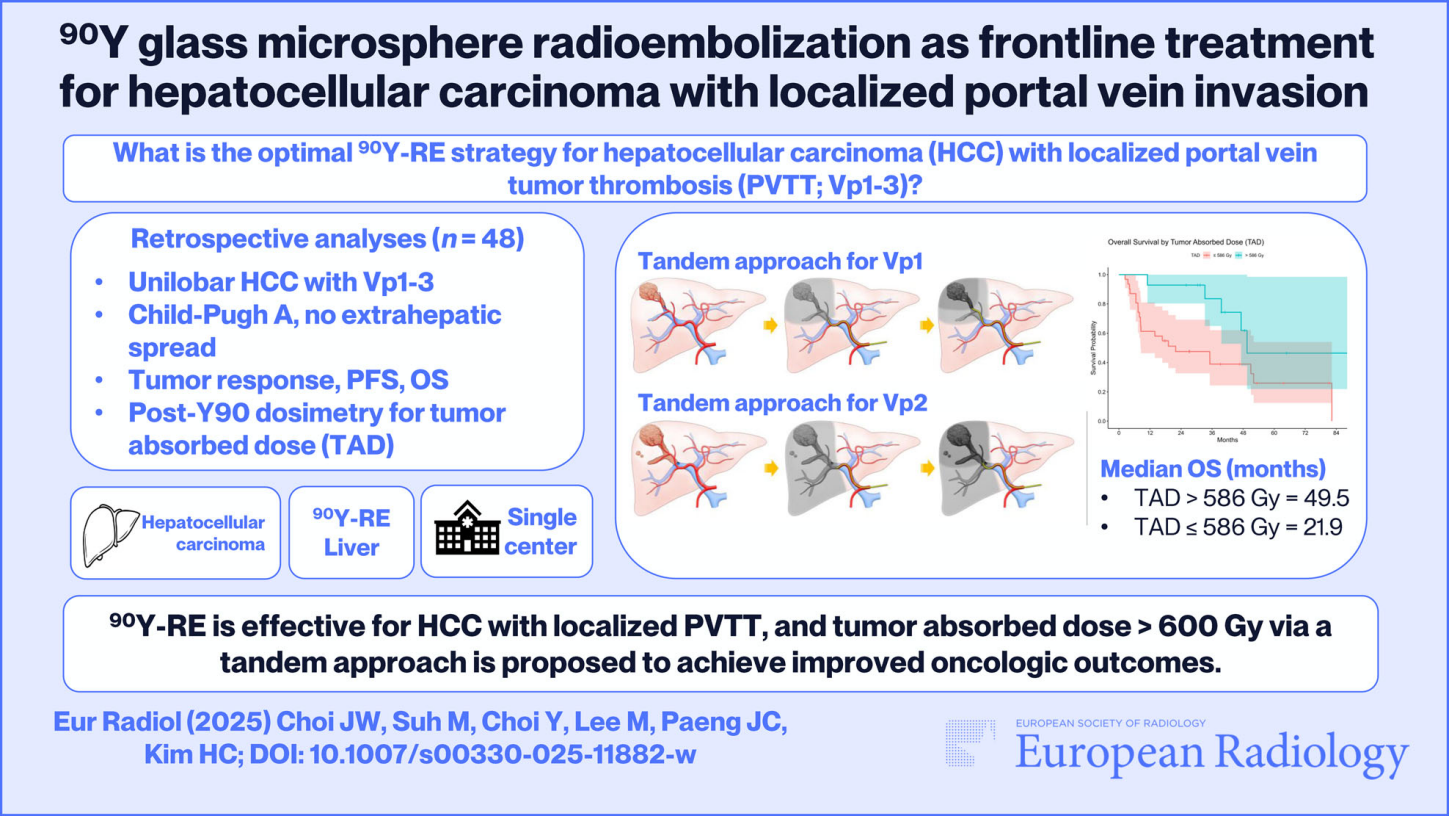

## Introduction

Hepatocellular carcinoma (HCC) with portal vein invasion (PVI) has traditionally been categorized within the realm of systemic treatments in the Barcelona Clinic Liver Cancer (BCLC) system [1]. However, in the management of HCC, where locoregional treatments are commonly utilized, their role in cases of localized PVI (i.e., Vp1 to Vp3 according to the Liver Cancer Study Group of Japan classification [2]) remains a subject of ongoing debate.

Accordingly, transarterial chemoembolization, transarterial chemoinfusion, external beam radiotherapy, and their combinations have been widely investigated [3–6]. Although there is limited literature providing outcomes stratified by the level of PVI and liver function, Choi et al reported a median overall survival (OS) of 32.7 months in patients with segmental PVI, Child-Pugh score ≤ 7, and no hepatic vein invasion after undergoing transarterial chemoembolization and chemoinfusion [7]. This finding underscores the potential of locoregional treatments in carefully selected patient populations, especially given that this study included patients treated between 2003 and 2012, a time when few systemic agents were effective in extending post-progression survival.

Yttrium-90 (Y90) radioembolization (^90^Y-RE) is now recognized as a standard-of-care in the management of HCC, and multiple clinical trials have evaluated its use for HCC with PVI. However, large-scale randomized controlled trials, including SARAH, SIRveNIB, and SORAMIC [8–10], enrolled patients with advanced HCC but failed to demonstrate the superiority of ^90^Y-RE or ^90^Y-RE combined with sorafenib over sorafenib alone. Retrospective analyses from high-volume institutions such as Northwestern University and the University of Milan have provided stratified survival outcomes for HCC with PVI [11, 12]. However, these studies involved heterogeneous patient populations with varying liver function and did not incorporate up-to-date dosimetry approaches. Consequently, the reported median OS for patients with segmental to sectional PVI ranged from 12 to 28 months [11, 12], a result less impactful in the current era of immune checkpoint inhibitors.

Recently, the DOSISPHERE-01 trial and post-hoc analyses of the SARAH trial demonstrated a direct correlation between tumor-absorbed dose (TAD) and oncologic outcomes [13, 14]. Subsequent studies have shown that when administered with an ablative dose, ^90^Y-RE can effectively devitalize the entire perfused tumor area [15, 16]. Despite these advancements, there is a lack of contemporary literature evaluating the efficacy of ^90^Y-RE using modern dosimetry approaches for HCC with localized PVI. This gap is substantial given the rapid evolution of the field and the potential for ablative embolization to provide meaningful outcomes in advanced but localized HCC.

To address this need, the present retrospective study was conducted to evaluate the clinical outcomes of ^90^Y-RE and the impact of dosimetry in patients with unilobar HCC and localized PVI (Vp1-3) who have preserved liver function.

## Materials and methods

### Patients

**Fig. 1:**
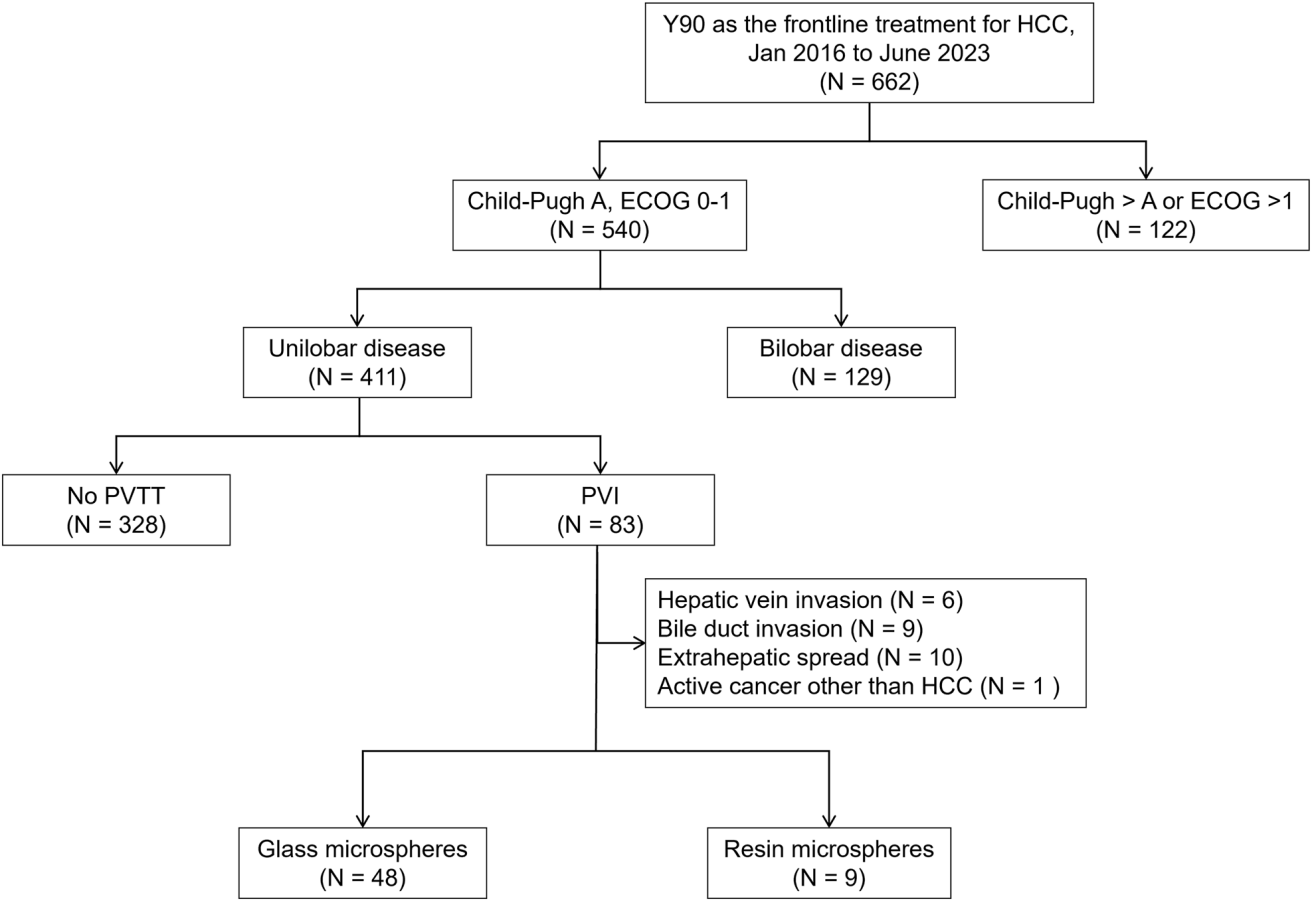
Selection of the study population. HCC, hepatocellular carcinoma; ECOG, Eastern Cooperative Oncology Group; PVI, portal vein tumor thrombosis

This retrospective study was approved by the Institutional Review Board, which waived the requirement for informed consent. Between January 2016 and June 2023, a total of 965 ^90^Y-RE procedures were performed for 873 patients at a tertiary referral hospital. Among these, 794 patients were diagnosed with HCC based on dynamic CT, MRI, or image-guided biopsy. A total of 662 treatment-naïve patients at the time of ^90^Y-RE were reviewed for this study. Patients with unilobar HCC, PVI confined to the tumor-bearing lobe (i.e., subsegmental to lobar PVI), Child-Pugh class A liver function, and an Eastern Cooperative Oncology Group (ECOG) performance status of 0 or 1 were initially included. The exclusion criteria comprised hepatic vein invasion, bile duct invasion, extrahepatic spread, and a history of active cancer within 2 years prior to ^90^Y-RE. Among 57 eligible patients with unilobar HCC and localized PVI, 48 who received Y‑90 glass microspheres were included in the final analysis (Fig. 1).

### Procedure

**Fig. 2:**
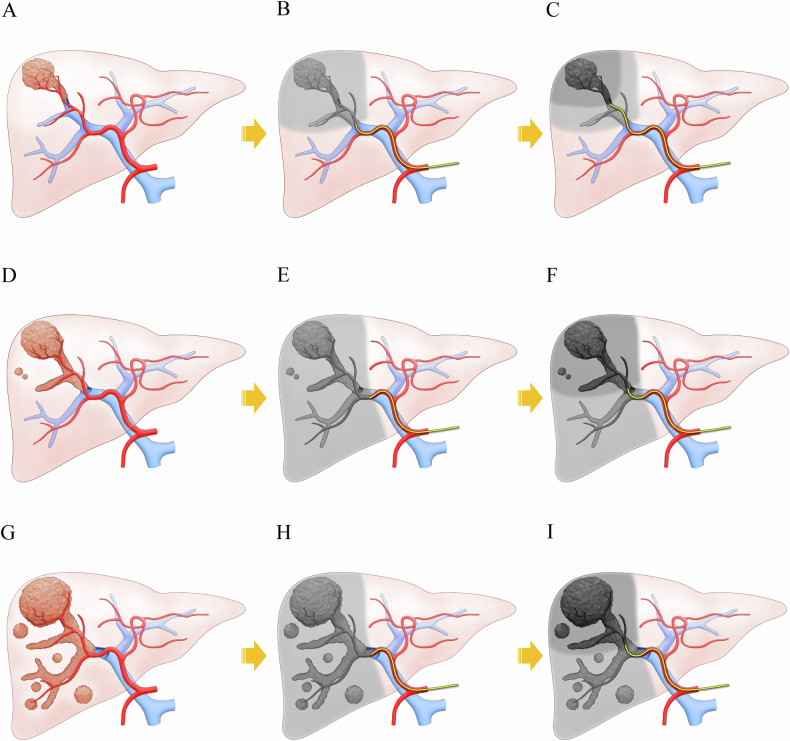
Tandem approach depending on the level of portal vein tumor thrombosis (PVI). **A** Hepatocellular carcinoma (HCC) with segment VIII PVI managed by Y90 radioembolization at **B** the right anterior sectional hepatic artery and **C** the segment VIII hepatic artery. **D** HCC with right anterior sectional PVI managed by Y90 radioembolization at **E** the right lobar hepatic artery and **F** the right anterior sectional hepatic artery. **G** HCC with right lobar PVI managed by Y90 radioembolization at **H** the right lobar hepatic artery and **I** the right anterior sectional hepatic artery

All procedures were conducted by one of three interventional radiologists (J.W.C., M.L., and H.C.K., with 7 to 24 years of experience in interventional radiology, respectively). Patients underwent planning angiography using cone-beam CT and 99mTc-macroaggregated albumin mapping, including planar imaging and SPECT-CT, prior to ^90^Y-RE. The tracer was injected into either the right or left lobar hepatic artery, and ^90^Y-RE was performed using Y90 glass microspheres (TheraSphere; Boston Scientific). Over the last decade, Y90 dosimetry has undergone significant advancements, leading to changes in treatment planning during the study period. In the first 2 years, radiation activity was prescribed to deliver approximately 120 Gy to the perfused volume using the single-compartment Medical Internal Radiation Dose (MIRD) method. Subsequently, dosimetry approaches were stratified based on the extent of PVI. For patients with subsegmental to sectional PVI (Vp1-2), treatment was performed with curative intent using either radiation segmentectomy (RS) or a tandem approach. RS was employed when both the tumor and PVI were clearly confined to a single segment, with doses exceeding 190 Gy during the early transition period and > 400 Gy thereafter. If the tumor and PVI extended beyond a single segment, a tandem approach—similar to a modified radiation lobectomy—was used. This approach delivered an ablative dose to the tumor-bearing segment(s) and a subablative dose to the portal venous territory one proximal level of the PVI (Fig. 2). For patients with lobar PVI (Vp3), either a tandem approach or simple lobar treatment was employed, depending on patient condition and treatment intent, ranging from near-curative to palliative. In tandem approaches, an ablative dose was delivered to the segment(s) with the highest tumor burden.

### Dosimetry analyses

Y90 PET-CT was conducted 1 day after the ^90^Y-RE procedure. Retrospective multi-compartment pre- and post-treatment dosimetry was performed using Simplicit90Y software (Mirada Medical). The segmentation process included defining the whole liver, perfused liver, tumor, and non-tumorous perfused liver based on diagnostic CT, MRI, or cone-beam CT. Multi-compartment MIRD and voxel-based dosimetry were then carried out using the segmented regions on the post-treatment Y90 PET-CT images.

### Clinical evaluation

Patients were advised to visit the hospital for follow-up at 1 month, 3 months, and every 3 months thereafter post-treatment. Decisions regarding curative conversion, such as surgical resection or liver transplantation, were made through a multidisciplinary team approach. The electronic medical records of the patients were retrospectively reviewed to identify post-procedural adverse events occurring within 90 days. Adverse events were graded based on the National Cancer Institute’s Common Terminology Criteria for Adverse Events (CTCAE), version 5.0. An interventional oncologist (H.C.K) evaluated the clinical course and follow-up imaging to assess tumor responses according to the modified Response Evaluation Criteria in Solid Tumors (mRECIST) and localized mRECIST [15, 17, 18]. The best tumor response observed between ^90^Y-RE and subsequent anti-cancer treatment or data censoring was recorded. The objective response rate (ORR) was calculated as the percentage of patients achieving either a partial or complete response (CR) (i.e., objective response) during this period. Survival data were obtained from the Ministry of the Interior and Safety of South Korea, which maintains a comprehensive survival database for all citizens, updated daily as of July 2024.

### Statistical analysis

Progression-free survival (PFS), hepatic progression-free survival (HPFS), and OS were estimated using the Kaplan–Meier method, and differences between the Vp1-2 and Vp3 groups were assessed with the log-rank test. PFS was defined as the time from ^90^Y-RE to death from any cause or disease progression at any site (e.g., liver, lung, bone, brain) based on mRECIST. HPFS was defined as the time from ^90^Y-RE to disease progression, specifically in the liver. For patients who achieved an objective response by mRECIST and localized mRECIST, the duration of response (DoR) was calculated as the time from the first documented objective response to the first recorded progressive disease or death from any cause, whichever occurred first. The median follow-up duration for evaluating the post-procedure clinical course was estimated using the reverse Kaplan–Meier method. In dosimetry analyses, the receiver operating characteristics (ROC) analysis was also conducted to determine what TAD determined by Y90 PET-CT predicted radiologic CR by localized mRECIST. The impact of the TAD, PVI level, tumor size (cm), tumor laterality (right vs. left), and serum alpha-fetoprotein level on OS was evaluated using univariate Cox proportional hazards regression. Variables with a *p*-value < 0.010 in the univariate analysis were included in the multivariate Cox proportional hazards regression. If the TAD was significantly associated with the OS, the cutoff value to obtain significantly longer OS was estimated by the minimum *p*-value approach.

A two-sided *p*-value of less than 0.05 was considered to indicate statistical significance. All statistical analyses were performed using the R statistical environment (version 4.3.2).

## Results

### Planning

The study population comprised 48 patients: 21 with Vp1-2 (44%, 21/48) and 27 with Vp3 (56%, 27/48). The largest tumor diameter was 7.1 ± 3.0 cm (mean ± standard deviation (SD)) (Table 1). The mean administered activity was 4.1 ± 1.6 GBq, corresponding to a mean absorbed dose of 283 ± 141 Gy to the perfused liver lobe, as calculated using the single-compartment MIRD method (Table 2). In terms of the treatment plan, RS, lobar treatment, and the tandem approach were performed for 2 patients (4.2%, 2/48), 8 patients (16.7%, 8/48), and 38 patients (79.2%, 38/48), respectively.

**Table 1:** Baseline characteristics of the 48 patients treated by radioembolization for hepatocellular carcinoma with localized portal vein invasion (PVI)

Variable	Value (%)
Gender	
Male	40 (83)
Female	8 (17)
Age (years)^a^	62 ± 12
Etiology of chronic liver disease	
Hepatitis B virus	38 (79)
Hepatitis C virus	3 (6)
Alcohol	2 (4)
Unknown	5 (10)
Child-Pugh class	
A	48 (100)
ECOG performance status	
0	45 (94)
1	3 (6)
Albumin-bilirubin grade	
1	32 (67)
2	16 (33)
Largest tumor diameter (cm)^a^	7.1 ± 3.0
Tumor laterality	
Right lobe	33 (69)
Left lobe	15 (31)
PVI level	
Vp1-2	21 (44)
Vp3	27 (56)
Alpha fetoprotein (ng/mL)^a^	15,734 ± 49,388
PIVKA-II (mAU/mL)^a^	8747 ± 30,250

*ECOG* Eastern Cooperative Oncology Group, *PIVKA-II* protein induced by vitamin K absence-II

^a^ Data are mean ± standard deviation

**Table 2:** Planning of radioembolization for hepatocellular carcinoma with localized portal vein invasion

Variable	Value (%)
Total liver volume (mL)^a^	1376 ± 284
Tumor volume (mL)^a^	251 ± 231
Treated liver volume (mL)^a^	724 ± 283
Total administered activity (GBq)^a^	4.1 ± 1.6
Days post-calibration	
2–5 days (1st week, Tuesday–Friday)	23 (48)
8–10 days (2nd week, Monday–Wednesday)	25 (52)
Mean absorbed dose by SC-MIRD (Gy)^a^	
Overall	283 ± 141
Vp1-2	275 ± 109
Vp3	288 ± 163
Lung shunt fraction (%)^a^	5.2 ± 4.2
Lung dose (Gy)^a^	10.1 ± 8.0

*C-MIRD* single-compartment medical internal radiation dose

^a^ Data are mean ± standard deviation

### Effectiveness

**Fig. 3:**
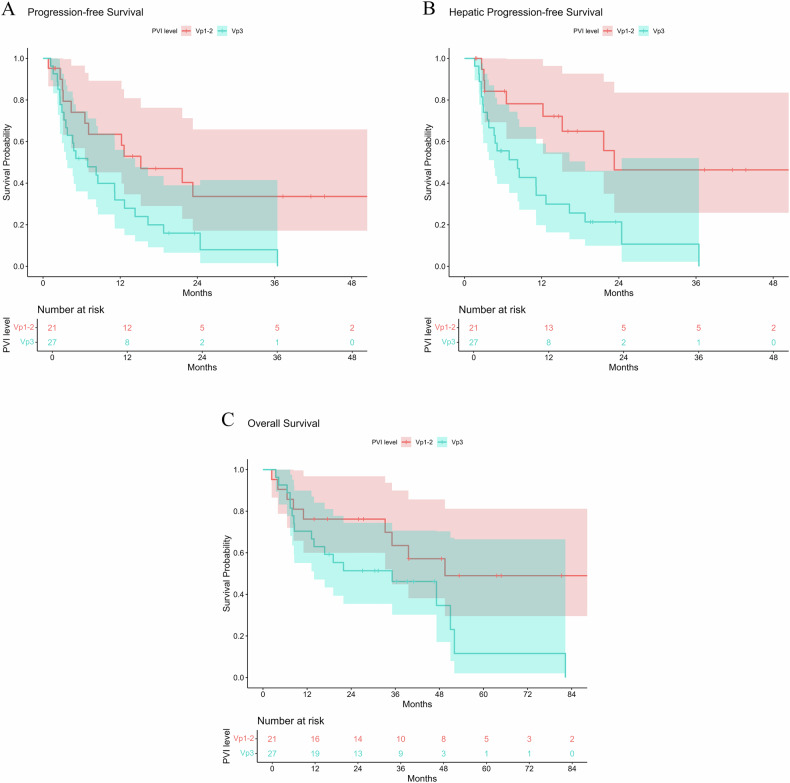
Post-radioembolization survival outcomes in patients with localized PVI. **A** Progression-free survival of the Vp1-2 group (median, 15.2 months; 95% confidence interval (CI), 4.4 to 23.3 months) and Vp3 group (median 6.9 months; 95% CI, 3.3 to 11.2 months) (*p* = 0.023). **B** Hepatic progression-free survival of the Vp1-2 group (median, 23.3 months; 95% CI, 12.2 to 23.3 months) and Vp3 group (median, 8.3 months; 95% CI, 3.6 to 12.7 months) (*p* = 0.004). **C** Overall survival of the Vp1-2 group (median, 49.5 months; 95% CI, 33.2 to 49.5 months) and Vp3 group (median, 35.1 months; 95% CI, 8.5 to 51.0 months) (*p* = 0.061)

The best radiologic tumor responses, as evaluated by mRECIST, were as follows: CR in 22 patients (46%, 22/48), partial response in 18 patients (38%, 18/48), stable disease in 5 patients (10%, 5/48), and progressive disease in 3 patients (6%, 3/48). According to localized mRECIST, the local tumor responses were: CR in 25 patients (52%, 25/48), partial response in 17 patients (35%, 17/48), stable disease in 5 patients (10%, 5/48), and progressive disease in 1 patient (2%, 1/48). The ORRs were 83% (40/48) by mRECIST and 88% (42/48) by localized mRECIST. The DoR for responders was 11.2 months (95% confidence interval (CI), 5.5–20.3 months) by mRECIST and 30.0 months (95% CI, 10.5–30.0 months) by localized mRECIST. The median PFS was 8.5 months (95% CI, 4.6–14.3 months), HPFS was 12.2 months (95% CI, 6.5–21.6 months), and OS was 47.2 months (95% CI, 19.1–52.1 months). The Vp1-2 group demonstrated significantly longer PFS (*p* = 0.023; hazard ratio (HR) = 0.462; 95% CI, 0.238–0.900) and HPFS (*p* = 0.004; HR = 0.338; 95% CI, 0.163–0.702) compared to the Vp3 group (Fig. 3). While the Vp1-2 group also exhibited longer OS (median, 49.5 months) than the Vp3 group (median, 35.1 months), the difference did not achieve statistical significance (*p* = 0.061; HR = 0.480; 95% CI, 0.222–1.035) in this study.

### Safety

Sixteen of the 48 patients (33%) experienced grade 3 or higher adverse events. The most common events were a decrease in lymphocyte count (17%, 8/48) and anemia (10%, 5/48) (Table 3). One case of radiation pneumonitis occurred following ^90^Y-RE with an estimated lung dose of 26.3 Gy. This patient developed fever and dyspnea 14 days post-treatment and was managed with steroid therapy. However, 5 weeks later, while tapering the steroids, the patient contracted SARS-CoV-2, developed acute respiratory distress syndrome, and experienced grade 4 elevations in aspartate aminotransferase and alanine aminotransferase levels. Despite intensive care unit management for 19 days, the patient expired. Aside from this case, no other patients died within 3 months of ^90^Y-RE.

**Table 3:** Adverse events following radioembolization for hepatocellular carcinoma with localized portal vein invasion

Terminology	CTCAE grade					Grade 3–4 (%)
	0	1	2	3	4	
Any adverse events^a^	2	10	20	15	1	16 (33)
Pneumonitis	47	0	0	1	0	1 (2)
Ascites	0	1	0	0	0	0 (0)
Fever	47	0	0	1	0	1 (2)
Fatigue	47	1	0	0	0	0 (0)
Nausea	40	8	0	0	0	0 (0)
Abdominal pain	23	19	5	1	0	1 (2)
Anemia	25	16	2	5	0	5 (10)
Lymphocyte count decreased	27	0	13	8	0	8 (17)
Platelet count decreased	26	17	2	3	0	3 (6)
Creatinine increased	47	1	0	0	0	0 (0)
Aspartate aminotransferase increased	35	6	5	1	1	2 (4)
Alanine aminotransferase increased	43	4	0	0	1	1 (2)
Alkaline phosphatase increased	32	14	2	0	0	0 (0)
Hypoalbuminemia	36	9	3	0	0	0 (0)
Blood bilirubin increased	36	6	3	3	0	3 (6)
Alkaline phosphatase increased	32	14	2	0	0	0 (0)

For individuals who underwent surgery prior to 90 days, only the data up to the point of surgery were analyzed

*CTCAE* common terminology criteria for adverse events, *SAE* serious adverse event

^a^ If a patient experienced multiple adverse events, the highest grade was marked

### Post-treatment clinical course

**Fig. 4:**
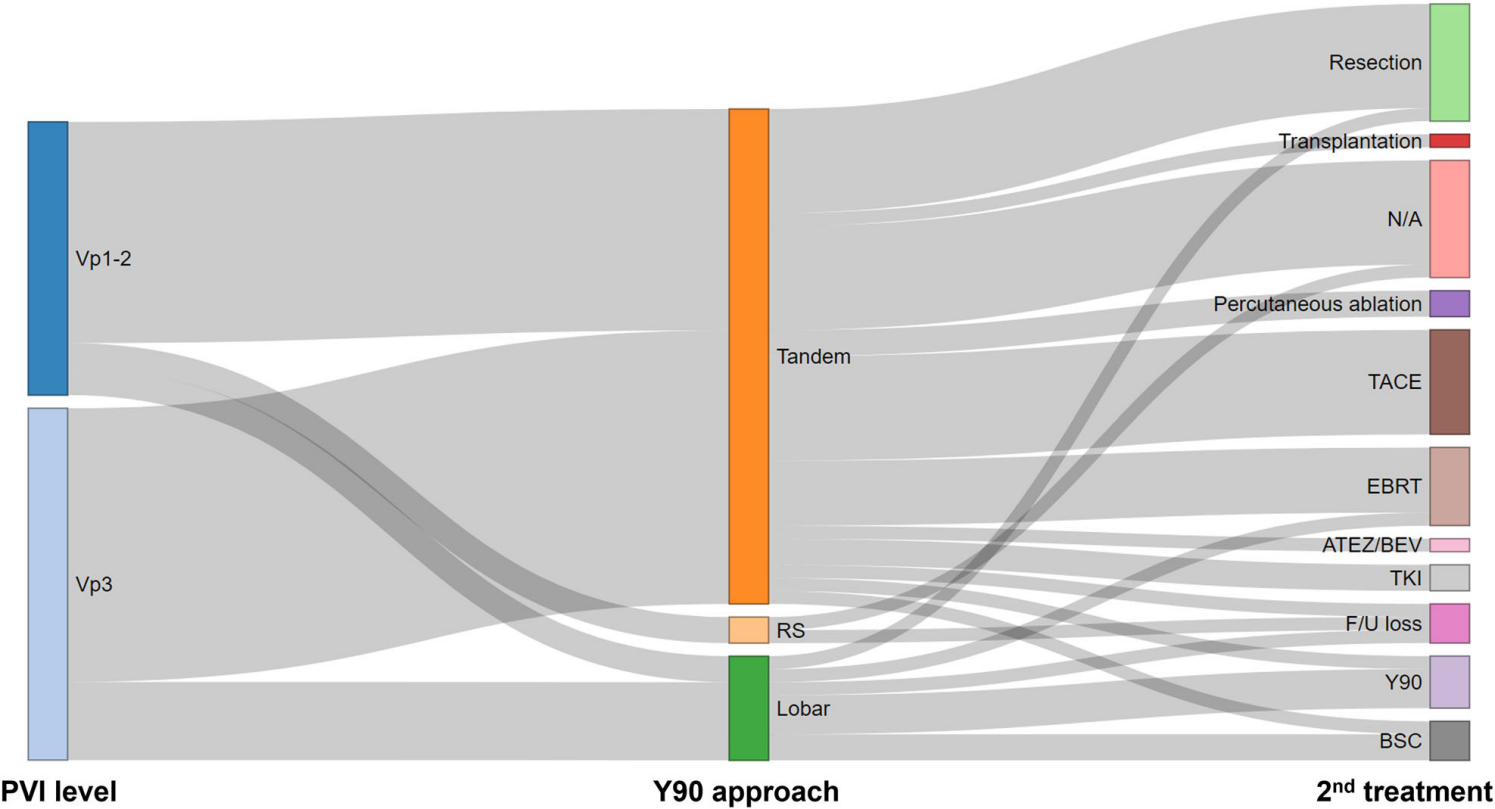
Sankey plot depicting the clinical course following radioembolization in patients with hepatocellular carcinoma and localized portal vein tumor thrombosis. PVI, portal vein tumor thrombosis; Vp1-2, segmental to sectional PVI; Vp3, lobar PVI; RS, radiation segmentectomy; N/A, not applicable due to the cancer-free status; TACE, transarterial chemoembolization; EBRT, external beam radiotherapy; ATEZ/BEV, atezolizumab plus bevacizumab; TKI, tyrosine kinase inhibitor; F/U, follow-up; BSC, best supportive care for palliation

Although OS was assessed for all patients, the clinical course was evaluated for 45 patients, excluding 3 who were lost to follow-up. The median follow-up duration for the clinical course was 46.6 months. Nine patients (20.0%, 9/45) did not receive any cancer-directed treatment after ^90^Y-RE due to a durable radiologic CR. Curative resection was conducted as the second treatment for 8 patients (17.8%, 8/45). Twenty patients (44.4%, 20/45) underwent locoregional treatments as their second-line therapy, including transarterial chemoembolization, repeat ^90^Y-RE, percutaneous ablation, or external beam radiotherapy (Fig. 4). During the follow-up periods, curative resection (*n* = 9) or liver transplantation (*n* = 1) was performed in 10 of the 48 patients (20.8%, 10/48). The median time from ^90^Y-RE to surgery was 3.8 months (interquartile range, 1.6 to 8.3 months). Among surgically treated patients, the median OS was 49.5 months. For patients with Vp1-2 (*n* = 6) who underwent surgery, the median OS was not reached, while for those with Vp3 (*n* = 4), the median OS was 19.1 months (95% CI, 13.2–27.4 months). Although the difference approached significance, it did not reach statistical significance (*p* = 0.058).

### Dosimetry analysis

**Fig. 5:**
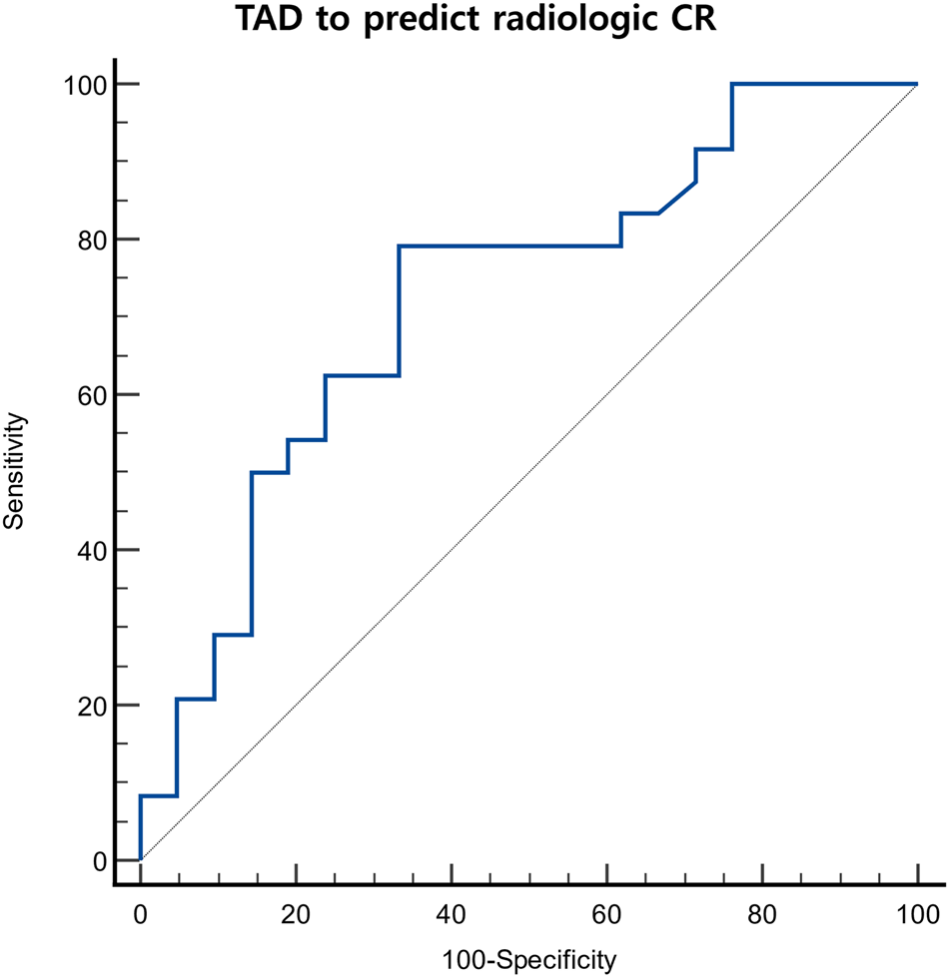
The receiver operating characteristics curve demonstrating the tumor-absorbed dose threshold and predictability of radiologic complete response by localized mRECIST (area under the curve, 0.725; 95% confidence interval, 0.572–0.848; *p* = 0.004)

**Fig. 6:**
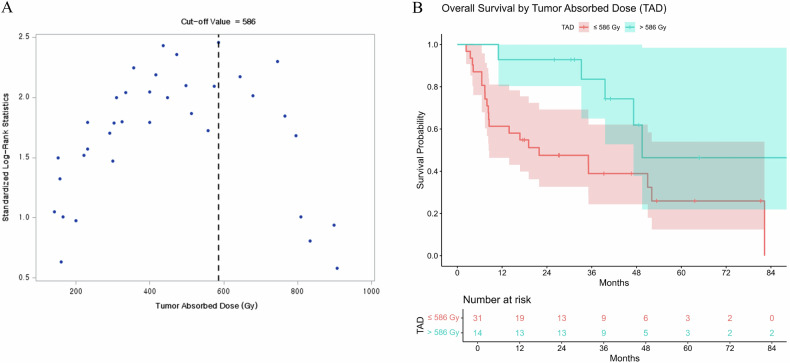
Post-radioembolization overall survival based on the proposed threshold dose of 586 Gy in patients with localized PVI. **A** Optimal cutoff determination using the minimum *p*-value approach. **B** Overall survival of the patients with a tumor-absorbed dose > 586 Gy and those with a tumor-absorbed dose ≤ 586 Gy

On pre-treatment SPECT-CT images, the tumor-to-normal liver uptake ratios (TNRs) were 3.7 ± 1.9 for patients with Vp1-2 and 2.6 ± 1.6 for those with Vp3. Notably, the TNR on Y90 PET-CT nearly doubled in patients with Vp1-2, where the tandem approach was frequently employed, reaching 7.4 ± 11.5. Correspondingly, the mean TAD was higher in patients with Vp1-2 (628 ± 377 Gy) compared to those with Vp3 (397 ± 248 Gy) (Table 4). The ROC analysis showed that the area under the curve was 0.725 (95% CI: 0.572–0.848; *p* = 0.004) (Fig. 5). Using a threshold of 335 Gy for the mean TAD, the sensitivity and specificity for predicting radiologic CR by localized mRECIST were 79% and 67%, respectively. To obtain higher specificity while maintaining acceptable sensitivity, a mean TAD threshold of 574 Gy provided 50% sensitivity and 86% specificity for predicting radiologic CR (positive predictive value, 80%). Post-treatment Y90 PET-CT analyses were performed for all but three patients who did not undergo Y90 PET-CT. Based on the univariate analyses, TAD (*p* = 0.031), PVI level (*p* = 0.067), and largest tumor size (*p* = 0.027) were included in the multivariate Cox proportional hazards analysis, and the TAD was identified as the only significant factor influencing OS (*p* = 0.032, hazard ratio = 0.862 per 100 Gy, 95% CI = 0.753 to 0.988). As a proposed threshold dose for HCC with localized PVI, 586 Gy was drawn as the cutoff with the lowest *p*-value (*p* = 0.021) (Fig. 6). The median OS of patients whose mTAD > 586 Gy and ≤ 586 Gy were 49.5 months and 21.9 months, respectively.

**Table 4:** Retrospective multi-compartment dosimetry

Variable	Value
Pre-treatment SPECT-CT analyses	
Tumor-to-normal liver uptake ratio	3.1 ± 1.8
Overall	3.7 ± 1.9
Vp1-2	2.6 ± 1.6
Post-treatment PET-CT analyses	
Tumor-to-normal liver uptake ratio	
Overall	5.1 ± 8.2
Vp1-2	7.4 ± 11.5
Vp3	3.3 ± 3.1
Mean tumor-absorbed dose (Gy)	
Overall	500 ± 329
Vp1-2	628 ± 377
Vp3	397 ± 248
D50 (Gy)^a^	
Overall	451 ± 314
Vp1-2	588 ± 368
Vp3	341 ± 214
D70 (Gy)^a^	
Overall	286 ± 234
Vp1-2	380 ± 287
Vp3	211 ± 149
V100 (%)^b^	
Overall	86 ± 18
Vp1-2	90 ± 13
Vp3	83 ± 21
Non-tumorous absorbed dose (Gy)	
Overall	153 ± 119
Vp1-2	150 ± 96
Vp3	155 ± 136

Data are mean ± standard deviation

^a^ D50 and D70, the cutoff dose value for the highest 50% and 70% of tumor voxels

^b^ V100, the percentage of tumor volume receiving more than 100 Gy

## Discussion

The present study demonstrated that ^90^Y-RE is both effective and safe for treating HCC with localized PVI in patients with preserved liver function. While direct comparisons with previous studies are challenging due to heterogeneity in patient characteristics, the median OS of 47.2 months (95% CI, 19.1–52.1 months) observed in this study is noteworthy. In the IMbrave150 trial, patients with macrovascular invasion had a median OS of 14.2 months (95% CI, 11.0–19.4 months) when treated with atezolizumab and bevacizumab [19]. In a low-risk subgroup (no PVI in the main portal vein, no bile duct invasion, and tumor infiltration ≤ 50% of the liver), the median OS was 22.8 months (95% CI, 19.1–24.9 months) with the same treatment combination [19]. While caution is necessary when comparing these results to the current study due to substantial differences in patient populations, the notable disparity in median OS suggests that ablative ^90^Y-RE may have the potential to be considered a frontline treatment option for HCC with localized PVI in patients with preserved liver function.

This study aimed to identify a threshold dose that could predict both a radiologic CR and a significant OS benefit following ^90^Y-RE. The analysis revealed that a mean TAD greater than 574 Gy yielded 50% sensitivity and 86% specificity for predicting radiologic CR by localized mRECIST. In other words, most patients who did not achieve CR received a TAD below 574 Gy. Additionally, the positive predictive value was 80%, suggesting that an 80% radiologic CR rate might be expected when the target tumor receives more than 574 Gy. Furthermore, OS was most significantly differentiated at a TAD threshold of 586 Gy. These findings are consistent with previous studies on ablative ^90^Y-RE for large HCCs (> 5 cm). For instance, Choi et al reported that tumors achieving CR by mRECIST received a mean TAD of 702.7 Gy (interquartile range, 383.4–830.8 Gy), whereas tumors with partial response or stable disease had a lower mean TAD of 281.2 Gy (interquartile range, 265.0–363.3 Gy) [18]. In aggregate, although further validation is required, a TAD threshold of approximately 600 Gy can be proposed to achieve improved oncologic outcomes in patients with localized PVI and preserved liver function undergoing ablative ^90^Y-RE.

Although the treatment approach in this study was somewhat heterogeneous due to its retrospective nature, the tandem approach was predominantly employed. This method aimed to deliver ablative doses to the primary tumor-bearing segment(s) while providing an angiosome-based therapeutic margin with a lower dose, balancing effective tumor control with short- and long-term safety considerations. HCC with PVI has a known risk of intrahepatic tumor spread, which typically follows the portal venous anatomy [20]. For instance, a peripherally located HCC with segmental PVI may initially spread along the affected segmental portal venous branch. As PVI progresses, intrahepatic metastases can develop along the sectional portal branch and eventually along the lobar portal vein. Based on this progression pattern, delivering Y90 microspheres to one level proximal to the visible PVI may help target micrometastases effectively. When the future liver remnant for curative resection is small, this approach may also promote hypertrophy of the contralateral lobe [21], making future resection feasible. Even in a worst-case scenario, where micrometastases have already occurred in the contralateral lobe before ^90^Y-RE, this strategy offers several advantages. By avoiding ablation of the entire tumor-bearing lobe and preserving liver function, it enables various subsequent treatment options to be explored.

This study has several limitations. First, it is subject to selection bias inherent to its retrospective design. Specifically, caution is required when comparing the outcomes of this study to previous research, as there is limited literature directly addressing the outcomes for the specific patient population of interest. The retrospective nature of the study also led to highly individualized post-procedure management and variations in treatment approaches over time, potentially limiting the generalizability of the results. Additionally, the dosimetry analyses aimed at determining a threshold dose for HCC with localized PVI were inconclusive. External validation with a different patient population is necessary to establish a widely accepted threshold dose for this group.

In conclusion, ^90^Y-RE is effective for HCC with localized PVI in patients with preserved liver function, and a mean TAD > 600 Gy is proposed to achieve improved oncologic outcomes.

## Abbreviations

CR: Complete response
CTCAE: Common terminology criteria for adverse events
DoR: Duration of response
ECOG: Eastern Cooperative Oncology Group
HCC: Hepatocellular carcinoma
HPFS: Hepatic progression-free survival
MIRD: Medical internal radiation dose
mRECIST: Modified response evaluation criteria in solid tumors
OS: Overall survival
PFS: Progression-free survival
PVI: Portal vein invasion
ROC: Receiver operating characteristics
RS: Radiation segmentectomy
TAD: Tumor-absorbed dose
TNR: Tumor-to-normal liver uptake ratio
Y90: Yttrium-90
Y90-RE: Yttrium-90 radioembolization

## References

[1] Reig M, Forner A, Rimola J et al (2022) BCLC strategy for prognosis prediction and treatment recommendation: the 2022 update. J Hepatol 76:681–693 34801630 10.1016/j.jhep.2021.11.018 PMC8866082

[2] Kudo M, Kitano M, Sakurai T, Nishida N (2015) General rules for the clinical and pathological study of primary liver cancer, nationwide follow-up survey and clinical practice guidelines: the outstanding achievements of the Liver Cancer Study Group of Japan. Dig Dis 33:765–770 26488173 10.1159/000439101

[3] Silva JP, Berger NG, Tsai S et al (2017) Transarterial chemoembolization in hepatocellular carcinoma with portal vein tumor thrombosis: a systematic review and meta-analysis. HPB (Oxford) 19:659–666 28552299 10.1016/j.hpb.2017.04.016

[4] He M, Li Q, Zou R et al (2019) Sorafenib plus hepatic arterial infusion of oxaliplatin, fluorouracil, and leucovorin vs sorafenib alone for hepatocellular carcinoma with portal vein invasion: a randomized clinical trial. JAMA Oncol 5:953–960 31070690 10.1001/jamaoncol.2019.0250 PMC6512278

[5] Lyu N, Wang X, Li JB et al (2022) Arterial chemotherapy of oxaliplatin plus fluorouracil versus sorafenib in advanced hepatocellular carcinoma: a biomolecular exploratory, randomized, phase III trial (FOHAIC-1). J Clin Oncol 40:468–480 34905388 10.1200/JCO.21.01963

[6] Yoon SM, Ryoo BY, Lee SJ et al (2018) Efficacy and safety of transarterial chemoembolization plus external beam radiotherapy vs sorafenib in hepatocellular carcinoma with macroscopic vascular invasion: a randomized clinical trial. JAMA Oncol 4:661–669 29543938 10.1001/jamaoncol.2017.5847 PMC5885246

[7] Choi JW, Kim HC, Lee JH et al (2017) Transarterial chemoembolization of hepatocellular carcinoma with segmental portal vein tumour thrombus. Eur Radiol 27:1448–1458 27516356 10.1007/s00330-016-4511-3

[8] Vilgrain V, Pereira H, Assenat E et al (2017) Efficacy and safety of selective internal radiotherapy with yttrium-90 resin microspheres compared with sorafenib in locally advanced and inoperable hepatocellular carcinoma (SARAH): an open-label randomised controlled phase 3 trial. Lancet Oncol 18:1624–1636 29107679 10.1016/S1470-2045(17)30683-6

[9] Chow PKH, Gandhi M, Tan SB et al (2018) SIRveNIB: selective internal radiation therapy versus sorafenib in Asia-Pacific patients with hepatocellular carcinoma. J Clin Oncol 36:1913–1921 29498924 10.1200/JCO.2017.76.0892

[10] Ricke J, Klumpen HJ, Amthauer H et al (2019) Impact of combined selective internal radiation therapy and sorafenib on survival in advanced hepatocellular carcinoma. J Hepatol 71:1164–1174 31421157 10.1016/j.jhep.2019.08.006

[11] Abouchaleh N, Gabr A, Ali R et al (2018) ^90^Y radioembolization for locally advanced hepatocellular carcinoma with portal vein thrombosis: long-term outcomes in a 185-patient cohort. J Nucl Med 59:1042–1048 29217739 10.2967/jnumed.117.199752

[12] Spreafico C, Sposito C, Vaiani M et al (2018) Development of a prognostic score to predict response to Yttrium-90 radioembolization for hepatocellular carcinoma with portal vein invasion. J Hepatol 68:724–732 29331342 10.1016/j.jhep.2017.12.026

[13] Garin E, Tselikas L, Guiu B et al (2021) Personalised versus standard dosimetry approach of selective internal radiation therapy in patients with locally advanced hepatocellular carcinoma (DOSISPHERE-01): a randomised, multicentre, open-label phase 2 trial. Lancet Gastroenterol Hepatol 6:17–29 33166497 10.1016/S2468-1253(20)30290-9

[14] Hermann AL, Dieudonne A, Ronot M et al (2020) Relationship of tumor radiation-absorbed dose to survival and response in hepatocellular carcinoma treated with transarterial radioembolization with ^90^Y in the SARAH study. Radiology 296:673–684 32602828 10.1148/radiol.2020191606

[15] Salem R, Johnson GE, Kim E et al (2021) Yttrium-90 radioembolization for the treatment of solitary, unresectable HCC: the LEGACY study. Hepatology 74:2342–2352 33739462 10.1002/hep.31819 PMC8596669

[16] Kim E, Sher A, Abboud G et al (2022) Radiation segmentectomy for curative intent of unresectable very early to early stage hepatocellular carcinoma (RASER): a single-centre, single-arm study. Lancet Gastroenterol Hepatol 7:843–850 35617978 10.1016/S2468-1253(22)00091-7

[17] Llovet JM, Lencioni R (2020) mRECIST for HCC: performance and novel refinements. J Hepatol 72:288–306 31954493 10.1016/j.jhep.2019.09.026 PMC12452114

[18] Choi JW, Jang MJ, Suh M, Kim HC (2024) Radiation major hepatectomy to selectively treat large unifocal hepatocellular carcinoma (RESCUE): protocol for an open-label, single-arm, single-center trial. J Vasc Interv Radiol 35:1221–1223 38723864 10.1016/j.jvir.2024.04.026

[19] Cheng AL, Qin S, Ikeda M et al (2022) Updated efficacy and safety data from IMbrave150: atezolizumab plus bevacizumab vs. sorafenib for unresectable hepatocellular carcinoma. J Hepatol 76:862–873 34902530 10.1016/j.jhep.2021.11.030

[20] Choi JY, Lee JM, Sirlin CB (2014) CT and MR imaging diagnosis and staging of hepatocellular carcinoma: Part I. Development, growth, and spread: key pathologic and imaging aspects. Radiology 272:635–654 25153274 10.1148/radiol.14132361 PMC4263631

[21] Vouche M, Lewandowski RJ, Atassi R et al (2013) Radiation lobectomy: time-dependent analysis of future liver remnant volume in unresectable liver cancer as a bridge to resection. J Hepatol 59:1029–1036 23811303 10.1016/j.jhep.2013.06.015 PMC5085290

